# An observational study on treatment regimens and effectiveness for psoriasis in real-world settings among 407 patients in Southeast China

**DOI:** 10.3389/fmed.2024.1328750

**Published:** 2024-01-29

**Authors:** Yuping Huo, Yike Huang, Tungchun Lee, Maoying Lin, Wenhung Chun

**Affiliations:** ^1^Department of Dermatology, Xiamen Chang Gung Hospital, Hua Qiao University, Xiamen, Fujian, China; ^2^Department of Dermatology, Linkou Chang-Gung Memorial Hospital, Taiwan, China

**Keywords:** psoriasis, real-world, registry, management, treatment response

## Abstract

**Introduction:**

While new targeted therapies have advanced psoriasis treatment, real-world data on comparative effectiveness is lacking. This study analyzed treatment regimens and response in an observational cohort, examining potential disparities between clinical trials and routine practice.

**Methods:**

Data from the Psoriasis Standardized Diagnosis and Treatment Center registry were analyzed. Patients with ≥1 follow-up were included. Treatment response was assessed using PASI 50/90 criteria. Factors associated with response were analyzed.

**Results:**

407 patients were included (46 first-time diagnosed, 361 previously diagnosed). A higher proportion of first-time diagnosed patients achieved treatment response than previously diagnosed (76.1% vs. 62.6%). Multivariable analysis identified factors associated with reduced response in previously treated patients.

**Conclusion:**

This real-world study found lower treatment response rates compared to clinical trials, especially in previously treated patients. Disparities highlight remaining unmet needs for psoriasis management. Combination and rotational strategies may improve outcomes in patients unresponsive to available therapies. Ongoing research on novel targets and pathways is warranted to address treatment gaps.

## Introduction

Psoriasis, a chronic immune-mediated inflammatory skin disorder, affects millions of individuals worldwide, imposing a substantial burden on both patients and society. Characterized by red, scaly plaques and patches on the skin, psoriasis not only impairs physical health but also has profound psychological and social implications (1, 2). In 2014, the World Health Organization acknowledged psoriasis as a significant non-communicable ailment, underscoring the distress associated with misdiagnosis, inadequate treatment, and the societal stigma linked to this condition (3).

The global prevalence of psoriasis varies between 0.09 and 11.4%, making it one of the most prevalent autoimmune skin disorders (4). While the exact prevalence varies across populations, regions, and ethnicities, it is estimated that approximately 125 million individuals worldwide are affected by psoriasis. This condition significantly impacts the quality of life, often leading to physical discomfort, itching, pain, and restricted mobility (1). Furthermore, the psychosocial consequences of psoriasis cannot be overlooked, as individuals may experience depression, anxiety, and social isolation due to the visible nature of the disease (5, 6). The economic burden associated with psoriasis is also substantial, encompassing direct medical costs, productivity losses, and impaired quality of life (7). This underscores the urgent need for effective treatment strategies.

Pathogenesis of psoriasis is complex, with genetics playing a pivotal role, particularly in early-onset plaque psoriasis (8, 9). This has been substantiated through various studies, including twin investigations, family-oriented analyses, and extensive population-level research, estimating heritability to be between 60 and 90% (9). Over 60 susceptibility loci have been identified through genome-wide association studies, known pathogenic pathways are related to antigen presentation, NF-kappa B signaling, and skin barrier function, etc. These findings imply a multifaceted interplay among T cells, dendritic cells, and keratinocytes as the likely drivers of psoriasis pathophysiology (8–11).

Over the past few decades, significant strides have been made in understanding the underlying immunopathology of psoriasis, leading to the development of targeted therapies (12, 13). Traditional treatment modalities such as topical corticosteroids, phototherapy, and systemic immunosuppressive agents have provided relief to many patients (14). However, the advent of biologic agents (biologics) and small molecule inhibitors targeting specific immune pathways has revolutionized psoriasis management (15). Biologics are derived from diverse natural sources, including human, animal, or microorganism origins, and can be manufactured using biotechnological approaches and advanced technologies. Unlike the majority of chemically synthesized drugs with well-defined structures, biologics typically exist as intricate mixtures that pose challenges in identification and characterization (16). These novel treatments which targeted to pathogenic pathways, including tumor necrosis factor (TNF) inhibitors, interleukin (IL)-17 and IL-23 inhibitors, and Janus kinase (JAK) inhibitors, have demonstrated remarkable efficacy and safety profiles in clinical trials (17–19). Their mode of action, which specifically targets key inflammatory mediators, not only improves skin symptoms but also addresses associated comorbidities such as psoriatic arthritis and cardiovascular risks (20, 21). Due to efficacy and safety of biologics, Adalimumab, Etanercept, Ustekinumab, Ixekizumab, Secukinumab were recommended as first-line therapy for psoriasis (22, 23).

Psoriasis is heterogeneous among patients and results in different inflammatory involvement and different clinical features (24). Variants interact not only with each other through “epistasis” but also with environmental factors, contributing to a multifactorial etiology. Additionally, it is plausible that some individuals with the condition may be the result of new mutations (25). An individual patient may vary from others in medical history and baseline clinical characteristics. These contribute to the complexity of psoriasis treatment. While the therapeutic landscape for psoriasis has undoubtedly advanced, several unmet needs persist (26). First, not all patients respond equally to available treatments, necessitating a personalized approach to therapy (27). Second, long-term safety and efficacy data for some of the newer agents are still evolving, and their impact on real-world populations remains to be fully understood (28). Third, treatment adherence and access to care can be challenging barriers, particularly in socioeconomically disadvantaged populations or regions with limited healthcare resources (29). Furthermore, the comparative effectiveness of different treatment regimens in diverse patient populations is not well-established, warranting comprehensive real-world studies.

In light of these considerations, the present clinical observational study aims to bridge the gap between controlled clinical trials and real-world treatment outcomes. While controlled trials provide valuable insights into the efficacy and safety of treatments, they often involve selected patient populations and strict protocols that may not fully represent the complexities of routine clinical practice (30). This study seeks to address this gap by assessing treatment regimens and their effectiveness in a diverse, real-world setting, encompassing a wide range of patient demographics, disease severities, and comorbidities.

To improve the overall diagnosis and treatment level of psoriasis in China, the National Clinical Research Center for Skin and Immune Disease launched a national collaborative project called the Psoriasis Standardized Diagnosis and Treatment Center (also named Psoriasis Center) project,^1^ in which the first national real-world big data collection platform, Psoriasis Center Registry, was established with more than 300 medical centers across China by 2022 (31). Clinical data of each psoriasis patient diagnosed or managed in the collaboration centers was recorded in the registry simultaneously with the medical documents.

As one of the collaboration center of the Registry, we were privileged to access partial data of the registry. By analyzing data, we intend to provide a comprehensive understanding of how different treatment modalities impact not only skin symptoms but also overall quality of life, comorbidities, and healthcare utilization. Furthermore, this study aims to identify factors influencing treatment adherence, access to care, and long-term outcomes, endeavoring to bridge the gap between controlled clinical trials and routine clinical practice, shedding light on the nuances of psoriasis management and providing valuable insights for optimizing patient care.

## Materials and methods

### Patients

The enrolled patients in this study was registered in the Psoriasis Standardized Diagnosis and Treatment Center project. Considering the fairness to all the collaboration centers of the project, data usage of the registry shall follow the policy of the project. The data was applied by the authors of this study to the board of the project and after the reviewing of our study protocol, the board will conduct a randomization of patient screening in the registry, and provide the corresponding data of the patients screened for this study. In this study, we included the patients with at least one follow-up after the baseline (when the patient was enrolled in the registry).

### Clinical data

Comprehensive clinical data were collected by the registry. For each patient, data included the baseline characteristics (gender, age, time of psoriasis diagnosis, etc.), medical history (smoking and alcohol in-taking, history of allergy, history of complicated chronic disease, duration of psoriasis, previous medication for psoriasis, etc.), baseline and evaluation during follow-ups (BSA, PASI, etc.), laboratory (CRP, ESR, CBC, liver and renal function, etc.), medication (localized and systematic medication, biologics, etc.). The data were structurally stored in the registry.

### Data cleaning

As the data was collected in a real-world setting, the variables may confronted with incompleteness. Considering the sample size and the feature of real-world study, the missing data were interpolated by means of mean imputation or set default as “No.” In addition, if an inconsistency among the primary variables (e.g., a patient reported as newly diagnosed but with previous psoriasis medication), the record was removed from the analysis. Patients with follow-up 12(±3) weeks after baseline were screened for analysis in this study.

### Treatment response

Response to treatments in psoriasis can be assessed using the PASI response 50, 90. If a patient achieved PASI response 90 or more, the treatment response was defined as obvious effectiveness, and PASI response 50 as effectiveness, while less than PASI response 50 as ineffectiveness. Ineffectiveness was also defined as unrespond to treatment, while obvious effectiveness and effectiveness were categorized as respond to treatment.

### Patient compliance

Patient compliance was evaluated on 1 month after the enrollment of the registry, inquiring the patients their actual medication and subsequent visits to those the attending physicians prescribed and scored 0 (completely incompliance) to 10 (full compliance) by the attending physicians of each patient. In this study, a score of 6 and more was defined as good compliance while a score less than 6 was defined as poor compliance.

### Statistical analysis

All statistical analyses were performed using R software^2^ and SPSS software (version 25.0, SPSS, IBM). Statistical description was number and proportion(%) for categorical variables and the mean ± standard deviation or median (interquartile range, IQR) for continuous variables, with normal or in-normal distribution, respectively. Student’s t-test or Mann–Whitney U tests for continuous variables and chi-square were used for categorical variables in univariate analysis. Logistic regression was used for multivariate analysis. Level of *p* < 0.05 was regarded as statistical significant in all analysis.

## Results

### Patient characteristic

After data cleaning, 407 patients were included in this analysis, involving 46 first-time diagnosed and 361 previously diagnosed.

### Response to the treatment

Treatment response rate was higher in first-time diagnosed patients compared to previously diagnosed (76.1% vs. 62.6%). There were no significant differences in baseline demographics like gender, smoking history, drug allergy history between the ineffective and effective groups. There were also no major differences in comorbidities, previous topical or systemic treatments, or baseline biologic use between the two groups. Previously diagnosed patients had significantly higher baseline BSA and PASI scores compared to first-time diagnosed patients. Most baseline lab values did not differ significantly between ineffective and effective groups, except hemoglobin was more likely to be abnormal in the ineffective group. The ineffective group had lower baseline PASI and BSA scores as well as lower PASI subdomain scores for trunk, arms and legs compared to the effective group. Table 1 listed the main characteristics and treatment response of first-time diagnosed and previously diagnosed patients, respectively. And detailed treatment regimens and abnormal findings in labs were listed in Supplementary Table S1.

**Table 1:** Characteristics and treatment response of first-time and previously diagnosed patients.

Variables		First-time diagnosed patients			Previously diagnosed patients		
		Unrespond to treatment	Respond to treatment	*p*	Unrespond to treatment	Respond to treatment	*p*
Gender	Male	4 (36.4%)	20 (57.1%)	0.229	108 (80.6%)	181 (80.1%)	0.907
	Female	7 (63.6%)	15 (42.9%)		26 (19.4%)	45 (19.9%)	
Compliance	Good	3 (27.2%)	24 (68.6%)	0.015	23 (17.2%)	165 (73.0%)	<0.001
	Poor	8 (72.8%)	11 (31.4%)		111 (82.8%)	61 (27.0%)	
Smoking history	Never	6 (54.5%)	17 (48.6%)	0.829	63 (47.0%)	128 (56.6%)	0.259
	Quit	1 (9.1%)	2 (5.7%)		17 (12.7%)	31 (13.7%)	
	Current Smoker	4 (36.4%)	16 (45.7%)		50 (37.3%)	65 (28.8%)	
	Occasional				0 (0.0%)	1 (0.4%)	
History of allergy	No	11 (100.0%)	32 (91.4%)	0.604	114 (85.1%)	187 (82.7%)	0.235
	Yes	0 (0.0%)	1 (2.9%)		7 (5.2%)	24 (10.6%)	
	Unknown	0 (0.0%)	2 (5.7%)		9 (6.7%)	14 (6.2%)	
Family history of psoriasis	Yes	2 (18.2%)	2 (5.7%)	0.434	20 (14.9%)	47 (20.8%)	0.392
	No	8 (72.7%)	30 (85.7%)		108 (80.6%)	170 (75.2%)	
	Unknown	1 (9.1%)	3 (8.6%)		6 (4.5%)	10 (4.4%)	
Diagnosed PsA at baseline	Yes	0 (0.0%)	12 (35.3%)	0.165	4 (3.0%)	8 (3.5%)	0.255
	No	5 (50.0%)	12 (35.3%)		57 (42.5%)	93 (41.2%)	
	Not Examined	3 (30.0%)	5 (14.7%)		29 (21.6%)	36 (15.9%)	
	To be Determined	2 (20.0%)	5 (14.7%)		27 (20.1%)	65 (28.8%)	
Previous biologics	Not Used				124 (92.5%)	207 (91.6%)	0.234
Age		44.55 ± 19.684	43.8 ± 15.791	0.91	42.98 ± 15.94	42.37 ± 17.2	0.734
Duration of psoriasis (Years)					12.14 ± 10.35	11.88 ± 10.14	0.822
BSA score at baseline		13.43 ± 14.69	26.74 ± 22.35	0.031	36.9 ± 31.6	47.8 ± 31.7	0.002
Head		3.18 ± 2.85	1.83 ± 2.69	0.184	4.55 ± 3.35	5.24 ± 3.19	0.056
Trunk		3.09 ± 2.62	5.43 ± 2.00	0.017	5.77 ± 3.26	6.85 ± 2.97	0.002
Arms		3.27 ± 2.76	4.89 ± 2.32	0.1	5.6 ± 3.23	6.43 ± 3.11	0.018
Legs		4.45 ± 2.54	4.91 ± 2.60	0.61	6.59 ± 3.25	7.26 ± 3.16	0.061
PASI Score at baseline		8.72 ± 10.52	10.90 ± 11.48	0.566	19.39 ± 17.11	25.57 ± 19.37	0.002

### Univariate analysis on variables with treatment response

To explore the clinical characteristics those might have impact on treatment response, in first-time diagnosed and previously diagnosed patients, respectively, univariate analysis was conducted to screen the variables with significant differences between groups of treatment response and unresponse. In first-time diagnosed patients, BSA score at baseline (13.43 ± 14.69; in treatment response group vs. 26.74 ± 22.35 in treatment unresponse group, *p* = 0.031) and trunk score (3.09 ± 2.62 vs. 5.43 ± 2.00, *p* = 0.017) were found to be statistical different in univariate analysis (Table 1). In previously diagnosed patients, statistical differences were found in BSA score at baseline (36.90 ± 31.68 vs. 47.86 ± 31.78, *p* = 0.002), trunk score (5.77 ± 3.26 vs. 6.85 ± 2.97, *p* = 0.002), arms score (5.60 ± 3.23 vs. 6.43 ± 3.11, *p* = 0.018) and PASI score at baseline (19.39 ± 17.11 vs. 25.57 ± 19.37, *p* = 0.002; Table 1).

### Biologics use

In first-time diagnosed patients, 16/46 (34.8%) patients received biologics. The response rate was not found statistical significant between the patients whether biologics were used. In previously diagnosed patients, 331/361 (91.7%) patients were biologics-naive at baseline, while the number reduced to 193 at the most recent follow-up. The response rate was higher in those received biologics (*p* = 0.009).

### Factors correlated to treatment response

Based on the provided multivariate analysis results, the key findings are: In the previously diagnosed group, baseline BSA had statistical significance on treatment effectiveness (*p* = 0.001). The four BSA subdomain scores (head, trunk, arms, legs) did not have statistical significance on treatment effectiveness. Baseline PASI score was also statistical significant (*p* < 0.001). Abnormal triglycerides (TG) had a negative trend with treatment effectiveness, but no statistical significance (*p* = 0.226). E antibody HBeAb positive had a very strong negative correlation with treatment effectiveness (*p* = 0.034). The constant term of the model was very large (*p* < 0.001), suggesting the model may need other variables added to improve fit. To explore the impact of treatment types on patients’ outcome, whether using of local treatment, systematic treatment and biologics were set as additional variables in multivariate analysis. Use of biologics was the independent factor for treatment response (*p* = 0.001). Due to the small sample size, in the first-time diagnosed group, no significant was found. Table 2 showed the result of the multivariate analysis of first-time diagnosed and previously diagnosed patients.

**Table 2:** Multivariate analysis of first-time diagnosed and previously diagnosed patients.

Variables	First-time diagnosed			Previously diagnosed		
	OR	95% CI	*p*	OR	95% CI	*p*
Abnormality in TG				0.464	0.134–1.609	0.226
Abnormality in HBeAb				0.155	0.028–0.872	0.034
BSA Score at Baseline	2.8	0.606–12.925	0.187	1.038	1.015–1.061	0.001
Head	0.194	0.01–3.865	0.283	1.006	0.861–1.174	0.942
Trunk	6.224	0.166–233.729	0.323	1.19	0.963–1.469	0.107
Arms	0.002	0–6.865	0.135	0.984	0.788–1.23	0.889
Legs	39.724	0.171–9,205	0.185	1.038	0.874–1.233	0.67
PASI Score at Baseline	18.266	0.02–16,803	0.404	0.865	0.81–0.924	<0.001
Local treatment	53.336	0.077–37,015	0.234	1.645	0.966–2.801	0.067
Systematic treatment				1.52	0.769–3.002	0.228
Biologics				2.345	1.412–3.896	0.001

### Subgroup analysis on patient compliance to treatment response

To explore the compliance to the treatment of the psoriasis patients in real-world settings of east China, and its impact on treatment response. Subgroup analysis was conducted on patients’ compliance (Table 1). The results showed that in first-time diagnosed patients, 27 of 46 (58.7%) patients were with good compliance, while 3 of 11 (27.2%) in unrespond-to-treatment group and 24 of 35 (68.6%) in respond-to-treatment group, which showed a significant statistical difference (*p* = 0.015) among the groups. In previously diagnosed patients, 188 of 460 (40.9%) of the patients were with good compliance, while 165 of 188 (87.8%) were response to treatment but only 61 of 172 (35.4%) with poor compliance reached treatment response (*p* < 0.001).

## Discussion

Psoriasis is an immune-mediated chronic inflammatory skin disease affecting approximately 2–4% of the population worldwide. In recent decades, there have been significant advances in psoriasis treatments, particularly with the development of biologic agents like tumor necrosis factor (TNF) inhibitors and interleukin (IL) inhibitors (32). However, despite the efficacy of new targeted therapies, some patients do not respond adequately or lose response over time. Real-world data on treatment outcomes are important to delineate remaining unmet needs.

Though previous randomized clinical trials such as resurface (33) had provided evidences on efficacy and safety of biologics, several real-world studies (27, 34) added more information on certain population and reported the treatment effectiveness in actual clinical settings, which may help the clinicians with comprehensive treatment strategy making.

This real-world study of 407 psoriasis patients found that a higher proportion of treatment-naïve patients (76.1%) achieved response compared to previously treated patients (62.6%). This lower response rate in the larger cohort of previously diagnosed patients highlights that psoriasis remains a challenge to manage in routine practice. Some potential factors include loss of efficacy over time, treatment non-adherence, and inconvenience of therapies leading to non-persistence (35). The disparity between clinical trial results and real-world outcomes emphasizes there is still room for improvement, especially for patients who have failed prior therapies.

The recent development of IL-17 and IL-23 inhibitors represent important additions to the psoriasis armamentarium, providing new options when TNF inhibitors are inadequate or intolerable. IL-17 inhibitors like secukinumab, ixekizumab and brodalumab have shown high levels of efficacy, with over 80% of patients achieving 75% reduction in Psoriasis Area and Severity Index (PASI) scores in trials (36). Similarly, the IL-23 inhibitors guselkumab and tildrakizumab have demonstrated superiority to the TNF-α inhibitor etanercept, with around 70% of patients reaching PASI 90 responses (37). Despite outstanding results, around 30% of patients still do not respond to these newest biologics, indicating further diversification of therapeutic targets is warranted.

This study’s real-world findings reinforce that psoriasis patients who have failed prior systemic treatments represent a difficult-to-treat population. Future directions may include exploring combination therapy and rotation strategies to improve outcomes in patients with inadequate responses. As our understanding of psoriasis immunopathogenesis expands, new cytokine targets and small molecule inhibitors are emerging as well. Agents that inhibit IL-36, IL-17C, and JAK–STAT signaling are currently under investigation for moderate-to-severe psoriasis (38). Continued efforts to identify key drivers of inflammation and develop drugs that target specific pathogenic pathways may further reduce refractory disease. In conclusion, while progress has been made, this real-world study highlights residual gaps in achieving satisfactory disease control for all psoriasis patients. Ongoing research and drug development are still required, especially for patients unresponsive to currently available treatments. The analysis in this study excluded patients for whom follow-up data could not be obtained, which may lead to an overestimation of treatment effectiveness. In fact, research on patient follow-up rates can indirectly reflect the overall compliance of the patient population with the treatment. However, because the analysis dataset in this study is based on the exclusion of patients for whom follow-up data could not be obtained, the overall patient follow-up rate was not calculated. This result should be noted and addressed in subsequent studies. The regression result of factors correlated to treatment response involved a large constant, which indicated the probability of existence of other potential variables. Literature search showed that demographic characteristics such as employment and marital status, exacerbation characteristics such as season and condition of the episode; patient satisfactory to previous treatment, nail involvement, IGA, adverse events could also be the factors of treatment response. More attentions on the factors from previous and current findings should be paid in future studies (39–41). Subgroup analysis of compliance on treatment response showed the compliance of patients were under satisfaction, while it showed significant impact on treatment response. Indications for using biologics in psoriasis are still not completely clear, and the factors influencing the efficacy of biologics are also not well understood. In this study, although no positive predictors of efficacy were found in the multivariate analysis, some variables showed significant differences in the univariate analysis. This suggests that certain factors may be associated with better outcomes, but the relationship is not strong enough to be detected in the multivariate model, likely due to the limited sample size. The trends observed in the univariate analysis warrant further investigation in future studies to elucidate the impact of these factors. Although the current study did not identify definitive predictors of biologics response, it provides clues on potential variables of interest that should be examined in larger, prospective cohorts. More research is needed to clarify the indications for biologics and to optimize treatment strategies based on patient and disease characteristics. Continuous analysis on registry data is also needed to track and evaluate if improvements in treatment strategy, adherence and outcome of the patients are made year after year. The findings lay the groundwork for future studies to develop personalized treatment algorithms to improve outcomes in psoriasis patients using biologic therapy.

Several limitations of this study should be acknowledged. First, the sample size was relatively small, which may limit the generalization of the findings. Second, there was some missing data for several participants which was imputed by means of the variables or set default as “No,” which may have influenced the results, the proportion of missing data of each variable was listed in Supplementary Table S2. Third, due to the complex of drug combinations among the patients and current small sample, subgroup analysis and regressions of previous medications and study result is not yet conducted. Finally, the follow-up of patients was suboptimal, with a significant loss to follow-up over time. This could introduce biases and affect the validity of the long-term outcomes. In summary, the small sample size, missing data, and poor patient follow-up are major limitations of this study that restrict the interpretability and generalizability of the results. Future studies with larger sample sizes, complete data, and adequate follow-up assessments are warranted to validate the findings from this preliminary study.

## Data availability statement

The original contributions presented in the study are included in the article/Supplementary material, further inquiries can be directed to the corresponding authors.

## Ethics statement

The studies involving humans were approved by the Ethic Committee of Xiamen Chang Gung Hospital (XMCGIRB2021023). The studies were conducted in accordance with the local legislation and institutional requirements. Written informed consent for participation in this study was provided by the participants’ legal guardians/next of kin.

## Author contributions

YuH: Conceptualization, Data curation, Writing – original draft, Writing – review & editing. YiH: Data curation, Formal analysis, Writing – original draft. TL: Data curation, Formal analysis, Writing – original draft. ML: Conceptualization, Formal analysis, Writing – review & editing. WC: Conceptualization, Formal analysis, Writing – review & editing.

## Footnotes

^1^ https://inf.news/en/health/6092eead00b62e8ef53223876e067751.html

^2^ http://www.R-project.org
