# Supplementary material for: An observational study on treatment regimens and effectiveness for psoriasis in real-world settings among 407 patients in Southeast China
Source: Front Med (Lausanne). 2024 Jan 29;11:1328750. doi: 10.3389/fmed.2024.1328750 (PMC10860679; doi:10.3389/fmed.2024.1328750)
Supplement: Supplementary file 1 [file Table_1.docx]

| **Variables** | | **First-time diagnosed patients** | | | **Previously diagnosed patients** | | |
| --- | --- | --- | --- | --- | --- | --- | --- |
|  |  | **Unresponsive to Treatment** | **Responded to Treatment** | ***P*** | **Unresponsive to Treatment** | **Responded to Treatment** | ***P*** |
| Previous Medication (Local) | Moisturizer/Emollient |  |  |  | 4 (3.0%) | 16 (7.1%) |  |
|  | Benvimod |  |  |  | 0 (0.0%) | 1 (0.4%) |  |
|  | Urea |  |  |  | 1 (0.7%) | 2 (0.9%) |  |
|  | Salicylic acid, urea, moisturizing/emollient |  |  |  | 0 (0.0%) | 1 (0.4%) |  |
|  | Glucocorticoids |  |  |  | 11 (8.2%) | 8 (3.5%) |  |
|  | Glucocorticoids, moisturizers/emollients |  |  |  | 5 (3.7%) | 6 (2.7%) |  |
|  | Glucocorticoids, urea, moisturizers/emollients |  |  |  | 1 (0.7%) | 1 (0.4%) |  |
|  | Glucocorticoids, salicylic acid, urea |  |  |  | 0 (0.0%) | 1 (0.4%) |  |
|  | Glucocorticoids, salicylic acid, urea, moisturizing/emollient |  |  |  | 0 (0.0%) | 1 (0.4%) |  |
|  | Glucocorticoids, retinoic acid |  |  |  | 0 (0.0%) | 1 (0.4%) |  |
|  | Glucocorticoids, Vitamin D3 |  |  |  | 7 (5.2%) | 4 (1.8%) |  |
|  | Glucocorticoids, Vitamin D3, Moisturizing/Emollient |  |  |  | 1 (0.7%) | 0 (0.0%) |  |
|  | Glucocorticoids, vitamin D3, calcineurin inhibitors |  |  |  | 1 (0.7%) | 0 (0.0%) |  |
|  | Glucocorticoids, vitamin D3, tar, moisturizing/emollient, calcineurin inhibitor |  |  |  | 1 (0.7%) | 0 (0.0%) |  |
|  | Glucocorticoids, Vitamin D3, Urea, Moisturizing/Emollient |  |  |  | 0 (0.0%) | 1 (0.4%) |  |
|  | Glucocorticoids, Vitamin D3, Urea, Tar, Moisturizing/Emollient |  |  |  | 0 (0.0%) | 1 (0.4%) |  |
|  | Glucocorticoids, vitamin D3, tretinoin, moisturizing/emollient |  |  |  | 0 (0.0%) | 1 (0.4%) |  |
|  | Vitamin A acid |  |  |  | 2 (1.5%) | 1 (0.4%) |  |
|  | Tretinoin, moisturizing/emollient |  |  |  | 0 (0.0%) | 1 (0.4%) |  |
|  | Vitamin D3 |  |  |  | 2 (1.5%) | 6 (2.7%) |  |
|  | Vitamin D3, moisturizer/emollient |  |  |  | 1 (0.7%) | 0 (0.0%) |  |
|  | Vitamin D3, urea |  |  |  | 0 (0.0%) | 1 (0.4%) |  |
|  | Vitamin D3, urea, moisturizing/emollient |  |  |  | 0 (0.0%) | 1 (0.4%) |  |
|  | Vitamin D3, retinoic acid |  |  |  | 0 (0.0%) | 2 (0.9%) |  |
|  | Phototherapy |  |  |  | 1 (0.7%) | 3 (1.3%) |  |
|  | cyclosporine |  |  |  | 1 (0.7%) | 4 (1.8%) |  |
|  | cyclosporine, retinoic acid |  |  |  | 1 (0.7%) | 0 (0.0%) |  |
|  | Methotrexate |  |  |  | 1 (0.7%) | 4 (1.8%) |  |
|  | Vitamin A acids |  |  |  | 4 (3.0%) | 12 (5.3%) |  |
|  | Vitamin A acid, phototherapy |  |  |  | 0 (0.0%) | 1 (0.4%) |  |
| Previous Biologics | Not Used |  |  |  | 124 (92.5%) | 207 (91.6%) | 0.234 |
|  | Adalimumab |  |  |  | 4 (3.0%) | 3 (1.3%) |  |
|  | Adalimumab, Etanercept |  |  |  | 1 (0.7%) | 0 (0.0%) |  |
|  | Secukinumab |  |  |  | 4 (3.0%) | 16 (7.1%) |  |
|  | Ixekizumab |  |  |  | 1 (0.7%) | 1 (0.4%) |  |
|  | Adalimumab |  |  |  | 0 (0.0%) | 4 (1.8%) |  |
|  | Adalimumab, secukinumab |  |  |  | 1 (0.7%) | 0 (0.0%) |  |
|  | Guselkumab |  |  |  | 0 (0.0%) | 1 (0.4%) |  |
|  | Secukinumab |  |  |  | 3 (2.2%) | 1 (0.4%) |  |
|  | Etanercept |  |  |  | 0 (0.0%) | 1 (0.4%) |  |
|  | Ixekizumab |  |  |  | 0 (0.0%) | 1 (0.4%) |  |
|  | Infliximab |  |  |  | 0 (0.0%) | 2 (0.9%) |  |
| Previous Medication (Systematic) | Moisturizer/Emollient |  |  |  | 3 (2.2%) | 6 (2.7%) |  |
|  | Moisturizer/Emollient, Calcineurin Inhibitor |  |  |  | 1 (0.7%) | 0 (0.0%) |  |
|  | Benvimod |  |  |  | 1 (0.7%) | 1 (0.4%) |  |
|  | Urea |  |  |  | 1 (0.7%) | 7 (3.1%) |  |
|  | Urea, moisturizing/emollient |  |  |  | 1 (0.7%) | 1 (0.4%) |  |
|  | Urea, Benvimod |  |  |  | 1 (0.7%) | 0 (0.0%) |  |
|  | Glucocorticoids |  |  |  | 11 (8.2%) | 19 (8.4%) |  |
|  | Glucocorticoids, moisturizers/emollients |  |  |  | 3 (2.2%) | 11 (4.9%) |  |
|  | Corticosteroids, Moisturizers/Emollientes, Calcineurin Inhibitors |  |  |  | 0 (0.0%) | 2 (0.9%) |  |
|  | Glucocorticoids, calcineurin inhibitors |  |  |  | 0 (0.0%) | 1 (0.4%) |  |
|  | Glucocorticoids, urea |  |  |  | 3 (2.2%) | 1 (0.4%) |  |
|  | Glucocorticoids, urea, moisturizers/emollients |  |  |  | 0 (0.0%) | 2 (0.9%) |  |
|  | Glucocorticoids, salicylic acid |  |  |  | 0 (0.0%) | 2 (0.9%) |  |
|  | Glucocorticoids, retinoic acid |  |  |  | 1 (0.7%) | 1 (0.4%) |  |
|  | Glucocorticoids, retinoic acid, moisturizing/emollient |  |  |  | 0 (0.0%) | 1 (0.4%) |  |
|  | Glucocorticoids, retinoic acid, salicylic acid |  |  |  | 0 (0.0%) | 1 (0.4%) |  |
|  | Glucocorticoids, Vitamin D3 |  |  |  | 9 (6.7%) | 9 (4.0%) |  |
|  | Glucocorticoids, Vitamin D3, Moisturizing/Emollient |  |  |  | 0 (0.0%) | 4 (1.8%) |  |
|  | Glucocorticoids, vitamin D3, moisturizing/emollient, calcineurin inhibitor |  |  |  | 1 (0.7%) | 0 (0.0%) |  |
|  | Glucocorticoids, vitamin D3, calcineurin inhibitors |  |  |  | 0 (0.0%) | 1 (0.4%) |  |
|  | Glucocorticoids, vitamin D3, urea |  |  |  | 0 (0.0%) | 1 (0.4%) |  |
|  | Glucocorticoids, Vitamin D3, Urea, Moisturizing/Emollient |  |  |  | 0 (0.0%) | 1 (0.4%) |  |
|  | Glucocorticoids, vitamin D3, salicylic acid |  |  |  | 1 (0.7%) | 1 (0.4%) |  |
|  | Glucocorticoids, vitamin D3, tretinoin, moisturizing/emollient |  |  |  | 0 (0.0%) | 1 (0.4%) |  |
|  | Vitamin A acid |  |  |  | 0 (0.0%) | 3 (1.3%) |  |
|  | Tretinoin, moisturizing/emollient |  |  |  | 0 (0.0%) | 1 (0.4%) |  |
|  | Vitamin D3 |  |  |  | 5 (3.7%) | 8 (3.5%) |  |
|  | Vitamin D3, moisturizer/emollient |  |  |  | 0 (0.0%) | 1 (0.4%) |  |
|  | Vitamin D3, urea, calcineurin inhibitor |  |  |  | 0 (0.0%) | 1 (0.4%) |  |
|  | Vitamin D3, retinoic acid, moisturizing/emollient |  |  |  | 0 (0.0%) | 1 (0.4%) |  |
|  | cyclosporine |  |  |  | 1 (0.7%) | 5 (2.2%) |  |
|  | cyclosporine, retinoic acid |  |  |  | 1 (0.7%) | 2 (0.9%) |  |
|  | Methotrexate |  |  |  | 4 (3.0%) | 9 (4.0%) |  |
|  | methotrexate, retinoic acid |  |  |  | 2 (1.5%) | 1 (0.4%) |  |
|  | Vitamin A acids |  |  |  | 10 (7.5%) | 24 (10.6%) |  |
|  | Vitamin A acids, leflunomide |  |  |  | 0 (0.0%) | 1 (0.4%) |  |
| Treatment Regimens (Local) used at baseline | No use | 6 (54.5%) | 12 (34.3%) | 0.229 | 92 (68.7%) | 136 (59.9%) | 0.096 |
|  | Moisturizer/Emollient | 1 (9.1%) | 1 (2.9%) |  | 3 (2.2%) | 6 (2.6%) |  |
|  | Moisturizer/Emollient, Calcineurin Inhibitor |  |  |  | 1 (0.7%) |  |  |
|  | Benvimod |  |  |  | 1 (0.7%) | 1 (0.4%) |  |
|  | Urea | 1 (9.1%) |  |  | 1 (0.7%) | 7 (3.1%) |  |
|  | Urea, Moisturizing/emollient |  |  |  | 1 (0.7%) | 1 (0.4%) |  |
|  | Urea, Benvimod |  |  |  | 1 (0.7%) |  |  |
|  | Glucocorticoids | 2 (18.2%) | 3 (8.6%) |  | 11 (8.2%) | 19 (8.4%) |  |
|  | Glucocorticoids, Moisturizers/emollients |  | 1 (2.9%) |  | 3 (2.2%) | 11 (4.8%) |  |
|  | Corticosteroids, Moisturizers/Emollientes, Calcineurin Inhibitors |  |  |  |  | 2 (0.9%) |  |
|  | Glucocorticoids, Calcineurin inhibitors |  |  |  |  | 1 (0.4%) |  |
|  | Glucocorticoids, Urea |  | 1 (2.9%) |  | 3 (2.2%) | 1 (0.4%) |  |
|  | Glucocorticoids, Urea, Moisturizers/emollients |  |  |  |  | 2 (0.9%) |  |
|  | Glucocorticoids, Salicylic acid |  |  |  |  | 2 (0.9%) |  |
|  | Glucocorticoids, Retinoic acid |  | 1 (2.9%) |  | 1 (0.7%) | 1 (0.4%) |  |
|  | Glucocorticoids, Retinoic acid, Moisturizing/emollient |  | 2 (5.7%) |  |  | 1 (0.4%) |  |
|  | Glucocorticoids, Retinoic acid, Salicylic acid |  |  |  |  | 1 (0.4%) |  |
|  | Glucocorticoids, Retinoic acid, Urea |  | 1 (2.9%) |  |  |  |  |
|  | Glucocorticoids, Vitamin D3 |  | 3 (8.6%) |  | 9 (6.7%) | 9 (4.0%) |  |
|  | Glucocorticoids, Vitamin D3, Moisturizing/Emollient |  | 3 (8.6%) |  |  | 4 (1.8%) |  |
|  | Glucocorticoids, Vitamin D3, Moisturizing/emollient, Calcineurin inhibitor |  |  |  | 1 (0.7%) |  |  |
|  | Glucocorticoids, Vitamin D3, Calcineurin inhibitors |  |  |  |  | 1 (0.4%) |  |
|  | Glucocorticoids, V D3, Urea |  | 1 (2.9%) |  |  | 1 (0.4%) |  |
|  | Glucocorticoids, Vitamin D3, Urea, Moisturizing/Emollient |  |  |  |  | 1 (0.4%) |  |
|  | Glucocorticoids, Vitamin D3, Salicylic acid |  | 2 (5.7%) |  | 1 (0.7%) | 1 (0.4%) |  |
|  | Glucocorticoids, Vitamin D3, Retinoic acid |  | 3 (8.6%) |  |  | 2 (0.9%) |  |
|  | Glucocorticoids, Vitamin D3, Tretinoin, Moisturizing/emollient |  | 1 (2.9%) |  |  | 1 (0.4%) |  |
|  | Tretinoin |  |  |  |  | 3 (1.3%) |  |
|  | Tretinoin, Moisturizing/emollient |  |  |  |  | 1 (0.4%) |  |
|  | Vitamin D3 | 1 (9.1%) |  |  | 5 (3.7%) | 8 (3.5%) |  |
|  | Vitamin D3, Moisturizer/emollient |  |  |  |  | 1 (0.4%) |  |
|  | Vitamin D3, Urea, Calcineurin inhibitor |  |  |  |  | 1 (0.4%) |  |
|  | Vitamin D3, Retinoic acid, Moisturizing/emollient |  |  |  |  | 1 (0.4%) |  |
| Biologics Used at Baseline | Adalimumab |  |  |  | 12 (9.0%) | 21 (9.3%) | 0.36 |
|  | Guselkumab |  |  |  | 0 (0.0%) | 1 (0.4%) |  |
|  | Other |  |  |  | 2 (1.5%) | 5 (2.2%) |  |
|  | Other tumor necrosis factor receptor antibody fusion proteins |  |  |  | 0 (0.0%) | 1 (0.4%) |  |
|  | Secukinumab |  |  |  | 33 (24.6%) | 81 (35.8%) |  |
|  | Not used |  |  |  | 84 (62.7%) | 109 (48.2%) |  |
|  | Ustekinumab |  |  |  | 0 (0.0%) | 1 (0.4%) |  |
|  | Etanerceptumab |  |  |  | 0 (0.0%) | 1 (0.4%) |  |
|  | Ixekizumab |  |  |  | 3 (2.2%) | 6 (2.7%) |  |
|  | Infliximab |  |  |  | 0 (0.0%) | 1 (0.4%) |  |
| Abnormal Lab Findings at Baseline | CRP |  |  |  | 1 (0.7%) | 9 (4.0%) |  |
|  | hs-CRP |  |  |  | 1 (0.7%) | 9 (4.0%) |  |
|  | ESR |  |  |  | 1 (0.7%) | 2 (0.9%) |  |
|  | WBC | 0 (0.0%) | 1 (6.3%) | 0.797 | 3 (2.2%) | 10 (4.4%) |  |
|  | RBC |  |  |  | 2 (1.5%) | 14 (6.2%) |  |
|  | Hb |  |  |  | 1 (0.7%) | 7 (3.1%) |  |
|  | PLT |  |  |  | 6 (4.5%) | 6 (2.7%) |  |
|  | TP |  |  |  | 4 (3.0%) | 8 (3.5%) |  |
|  | G |  |  |  | 4 (3.0%) | 12 (5.3%) |  |
|  | Alb |  |  |  | 1 (0.7%) | 4 (1.8%) |  |
|  | AST |  |  |  | 9 (6.7%) | 16 (7.1%) |  |
|  | ALT |  |  |  | 4 (3.0%) | 7 (3.1%) |  |
|  | STB |  |  |  | 3 (2.2%) | 7 (3.1%) |  |
|  | DBil |  |  |  | 3 (2.2%) | 2 (0.9%) |  |
|  | BUN |  |  |  | 1 (0.7%) | 5 (2.2%) |  |
|  | Scr |  |  |  | 3 (2.2%) | 5 (2.2%) |  |
|  | TG |  |  |  | 1 (0.7%) | 3 (1.3%) |  |
|  | TC |  |  |  | 6 (4.5%) | 6 (2.7%) |  |
|  | HDL-C |  |  |  | 4 (3.0%) | 8 (3.5%) |  |
|  | LDL-C |  |  |  | 4 (3.0%) | 13 (5.8%) |  |
|  | Fasting Glucose |  |  |  | 4 (3.0%) | 8 (3.5%) |  |
|  | P2hPG |  |  |  | 2 (1.5%) | 6 (2.7%) |  |
|  | GHb |  |  |  | 1 (0.7%) | 2 (0.9%) |  |
|  | Cutaneous Fungal Microscopy |  |  |  | 1 (0.7%) | 2 (0.9%) |  |
|  | HBsAb |  |  |  | 2 (1.5%) | 1 (0.4%) |  |
|  | HBeAg |  |  |  | 10 (7.5%) | 29 (12.8%) |  |
|  | HBeAb |  |  |  | 0 (0.0%) | 2 (0.9%) |  |
|  | HBcAb |  |  |  | 6 (4.5%) | 4 (1.8%) |  |
|  | HBV DNA |  |  |  | 8 (6.0%) | 4 (1.8%) |  |
|  | HCV IgM |  |  |  | 0 (0.0%) | 1 (0.4%) |  |
|  | Syphilis serum reagin |  |  |  | 0 (0.0%) | 2 (0.9%) |  |

**Table S1 Treatment regimens, abnormal lab findings and treatment response of first-time and previously diagnosed patients**
