# Supplementary material for: An observational study on treatment regimens and effectiveness for psoriasis in real-world settings among 407 patients in Southeast China
Source: Front Med (Lausanne). 2024 Jan 29;11:1328750. doi: 10.3389/fmed.2024.1328750 (PMC10860679; doi:10.3389/fmed.2024.1328750)
Supplement: Supplementary file 2 [file Table_2.docx]

**Table S2** **Missing Imputation Rules and Missing Rates of Variables**

| **Variables** | **Missing Imputation Rule** | **Missing (%)** |
| --- | --- | --- |
| Gender | No missing data allowed | 0.0% |
| Compliance | No missing data allowed | 0.0% |
| Smoking History | Default as "No" | 0.1% |
| History of Allergy | Default as "No" | 0.1% |
| Family History of Psoriasis | Default as "No" | 1.5% |
| Diagnosed PsA at Baseline | No missing data allowed | 0.0% |
| Previous biologics | No missing data allowed | 0.0% |
| Age | No missing data allowed | 0.0% |
| Duration of Psoriasis (Years) | No missing data allowed | 0.0% |
| BSA Score at Baseline | No missing data allowed | 0.0% |
| Head | No missing data allowed | 0.0% |
| Trunk | No missing data allowed | 0.0% |
| Arms | No missing data allowed | 0.0% |
| Legs | No missing data allowed | 0.0% |
| PASI Score at Baseline | No missing data allowed | 0.0% |
| Previous Medication (Local) | No missing data allowed | 0.0% |
| Previous Biologics | No missing data allowed | 0.0% |
| Previous Medication (Systematic) | No missing data allowed | 0.0% |
| Treatment Regimens (Local) used at baseline | No missing data allowed | 0.0% |
| Biologics Used at Baseline | No missing data allowed | 0.0% |
| Abnormal Lab Findings at Baseline | Default as "No" | 24.6% |
